# Olink Proteomics in Heart Failure: A Comprehensive Review of Applications From Biomarker Discovery to Pathophysiological Insights and Clinical Implementation

**DOI:** 10.31083/RCM45958

**Published:** 2026-01-22

**Authors:** Zhaolei Zheng, Zheng Gong, Jianhua Gu

**Affiliations:** ^1^Department of Emergency Medicine, Qilu Hospital of Shandong University, 250012 Jinan, Shandong, China; ^2^Shandong Provincial Clinical Research Center for Emergency and Critical Care Medicine, Institute of Emergency and Critical Care Medicine of Shandong University, Chest Pain Center, Qilu Hospital of Shandong University, 250012 Jinan, Shandong, China; ^3^Key Laboratory of Emergency and Critical Care Medicine of Shandong Province, Key Laboratory of Cardiopulmonary-Cerebral Resuscitation Research of Shandong Province, Shandong Provincial Engineering Laboratory for Emergency and Critical Care Medicine, Shandong Key Laboratory: Magnetic Field-free Medicine & Functional Imaging, Qilu Hospital of Shandong University, 250012 Jinan, Shandong, China

**Keywords:** heart failure, proteomics, biomarkers, precision medicine, translational medical research

## Abstract

Heart failure (HF) remains a global health challenge characterised by significant clinical heterogeneity, necessitating more precise tools for diagnosis and risk stratification. Olink proteomics, a high-throughput platform based on proximity extension assays (PEAs), has emerged as a powerful technology for exploring the molecular landscape of HF. Despite a growing number of studies utilising this platform, a comprehensive synthesis of its clinical and mechanistic contributions is still lacking. This review systematically examines the application of Olink proteomics across the HF continuum. We synthesised evidence regarding its role in biomarker discovery for early detection and prognosis, its ability to dissect key pathophysiological pathways such as inflammation and fibrosis, and its emerging potential to guide precision medicine. By critically evaluating technological advances, current challenges, and future directions, this review concludes that Olink proteomics is pivotal for transitioning HF management from a phenotype-driven to a mechanism-based paradigm, paving the way for targeted therapies and improved patient outcomes.

## 1. Introduction

Heart failure (HF) is a global pandemic affecting more than 64 million individuals worldwide, with the prevalence projected to increase substantially because of population aging and improved survival following acute cardiovascular events [1]. Despite significant therapeutic advances, HF remains associated with poor prognosis, with 5-year mortality rates approaching 50% following hospitalization [2, 3]. The heterogeneous nature of HF, encompassing diverse etiologies, pathophysiological mechanisms, and clinical phenotypes, poses fundamental challenges for diagnosis, risk stratification, and treatment optimization.

Traditional approaches to HF management rely heavily on clinical assessment, imaging modalities, and a limited repertoire of biomarkers, primarily natriuretic peptides [2, 4]. However, these conventional tools provide incomplete insights into the complex molecular landscape underlying HF pathogenesis and progression [5]. The emergence of high-throughput proteomic technologies has opened unprecedented opportunities to comprehensively investigate the circulating proteome, offering the potential for an enhanced understanding of disease mechanisms and the identification of novel therapeutic targets [6, 7, 8].

**Fig. 1.**
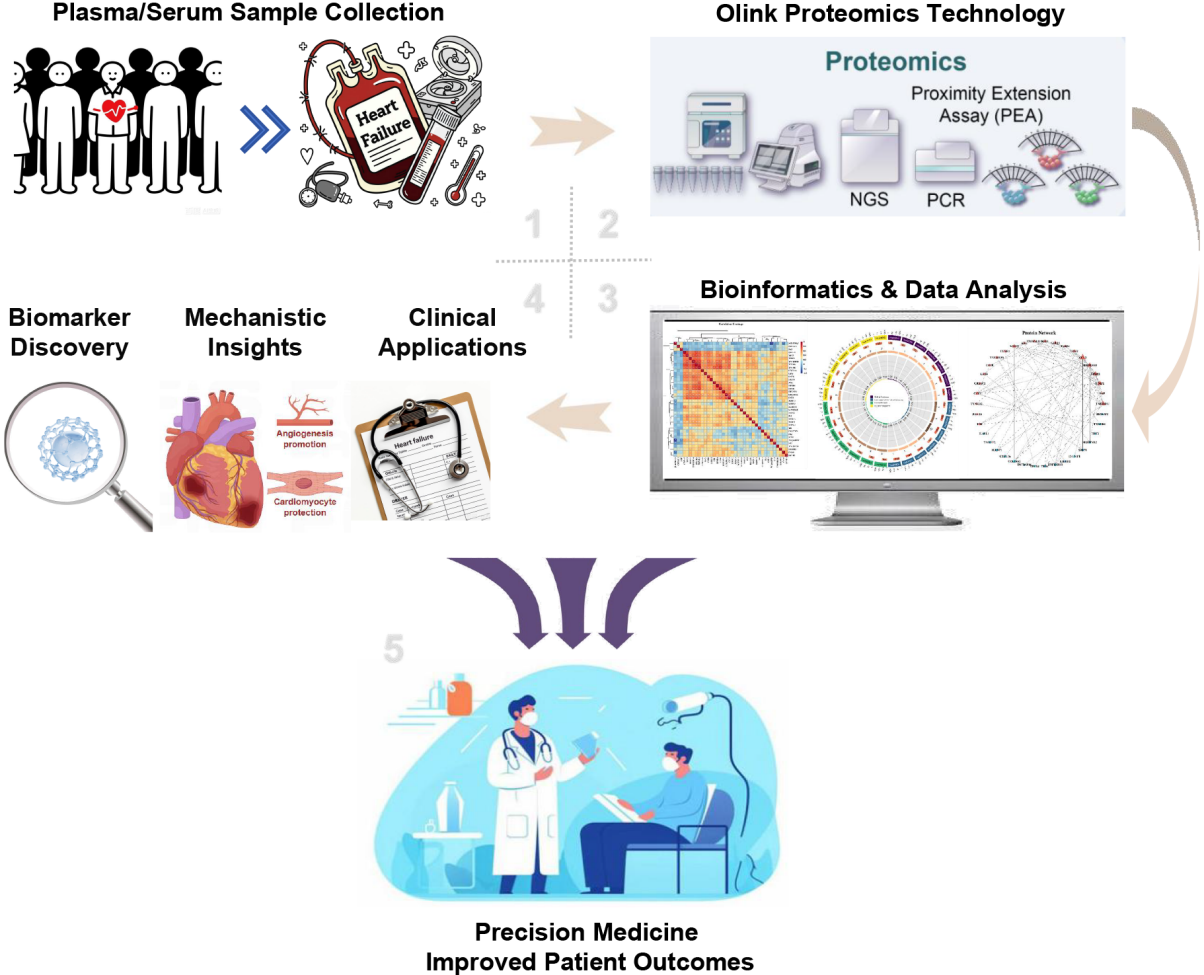
**The comprehensive workflow of Olink proteomics in heart failure (HF) research and clinical translation**. The process begins with the (1) collection of plasma or serum samples from diverse patient populations, including community-based cohorts and individuals with HF. (2) These samples are then analyzed using Olink’s Proximity Extension Assay (PEA), a high-throughput technology for protein quantification. (3) The resulting high-dimensional proteomic data is subjected to comprehensive bioinformatic analysis, such as expression profiling (heatmaps), network analysis, and pathway enrichment analysis, to identify key biological signals. (4) The insights derived are applied across three primary domains: (A) Biomarker Discovery, identifying novel proteins for patient risk stratification and prognosis; (B) Mechanistic Insights, to elucidate the underlying pathophysiological pathways of HF, including inflammation, fibrosis, and cardiac remodeling; and (C) Clinical Applications, for refined molecular phenotyping of patients and guiding therapeutic development. (5) The culmination of this workflow is the ultimate goal of transitioning towards precision medicine, enabling personalized treatment strategies and ultimately improving outcomes for patients with heart failure.

Among the available proteomic platforms, Olink technology, which is based on proximity extension assay (PEA) principles, has gained particular prominence in cardiovascular research [9, 10]. This innovative approach combines the specificity of dual antibody recognition with the sensitivity of DNA amplification, enabling simultaneous quantification of hundreds to thousands of proteins from minimal sample volumes. The technology’s unique of this technology address the critical limitations of conventional proteomic methods, making it particularly suitable for large-scale clinical studies and biobanking initiatives. A comprehensive workflow illustrating the application of Olink proteomics in HF research, from sample analysis to clinical translation, is depicted in Fig. 1.

This comprehensive review aims to synthesize current evidence regarding Olink proteomics applications in HF research, providing critical analysis of technological capabilities, clinical utility, and implementation challenges. We systematically examine the platform’s contributions to biomarker discovery, mechanistic understanding, and therapeutic development, while addressing controversies and future directions in the field.

## 2. Technological Foundations and Methodological Considerations

### 2.1 Principles of Olink Proximity Extension Assay Technology

The Olink platform represents a paradigm shift in protein quantification methodology, leveraging the PEA principle to achieve unprecedented multiplexing capability with exceptional analytical performance [9, 10]. The fundamental mechanism involves paired oligonucleotide-labeled antibodies that bind to target proteins. When both antibodies bind in close proximity, typically within 2–3 nanometers, the conjugated oligonucleotides hybridize and extend through DNA polymerase activity, creating an amplifiable template for subsequent detection via quantitative PCR or next-generation sequencing.

This dual-recognition strategy has confers multiple advantages over conventional immunoassays. The requirement for the simultaneous binding of two antibodies dramatically reduces the background signal and cross-reactivity, whereas DNA amplification enables the detection of proteins at femtomolar concentrations [9]. Recent technological iterations have expanded from focused 92-protein panels to comprehensive platforms encompassing more than 5000 proteins, with continuous improvements in antibody selection, oligonucleotide design, and normalization algorithms enhancing analytical performance.

Validation studies in cardiovascular cohorts have demonstrated excellent correlations between Olink measurements and established immunoassays, with correlation coefficients exceeding 0.9 for most validated targets [10]. The platform’s dynamic range, spanning 10 logs of protein concentration, surpasses that of conventional methods while maintaining coefficients of variation below 15% for the majority of analytes. These performance characteristics have been consistently reproduced across multiple laboratories, supporting the robustness of the technology for multicentre studies.

### 2.2 Evolution and Development in Cardiovascular Applications

The application of Olink technology in cardiovascular research has evolved substantially since its introduction. Initial studies in chronic myeloid leukemia patients demonstrated the feasibility of multiplexed proteomic profiling, successfully identifying treatment-related changes in protein expression patterns [9]. Translation to cardiovascular disease revealed the platform’s particular utility for detecting low-abundance inflammatory mediators and signaling molecules crucial to disease pathogenesis.

In acute coronary syndrome research, early applications of Olink Proseek technology revealed novel associations between inflammatory proteins and recurrent events [10]. Analysis of 29 immune and inflammatory proteins revealed that CXCL1, CD84, and TNF receptor superfamily member 10a (TNFRSF10A) independently predicted early recurrent acute coronary syndrome, demonstrating the ability of this technology to identify clinically relevant biomarkers beyond traditional risk factors. These findings establish a the foundation for broader application in HF research.

This technology has proven especially valuable in addressing the complexity of HF heterogeneity. Unlike traditional single-biomarker approaches, Olink enables comprehensive profiling of multiple biological pathways simultaneously, capturing the multifaceted nature of HF pathophysiology. Studies comparing different HF phenotypes have revealed distinct proteomic signatures, challenged traditional classification schemes and suggesting opportunities for molecularly-guided therapeutic strategies [11].

### 2.3 Comparison With Traditional Screening Methods

While traditional screening methods such as electrocardiography (ECG) and established biomarkers such as natriuretic peptides remain cornerstones of cardiac assessment [11, 12], Olink proteomics offers distinct advantages for deep molecular phenotyping and discovery. Its value is best understood when compared directly with that of other protein analysis technologies.

Versus Mass Spectrometry (MS)-based Proteomics: MS provides unparalleled depth for unbiased discovery and the ability to identify post-translational modifications [13, 14]. However, in the context of plasma proteomics for cardiovascular disease, MS faces significant challenges with the vast dynamic range of protein concentrations, where highly abundant proteins like albumin can mask the detection of low-abundance (but biologically crucial) cytokines and growth factors [15, 16]. Olink’s antibody-based affinity approach bypasses this issue, demonstrating superior sensitivity for many low-abundance signaling proteins without the need for extensive sample depletion or fractionation, thus enhancing throughput for large clinical cohorts [9, 17].

Versus Traditional Multiplex Immunoassays (e.g., Luminex, ELISA): Standard immunoassays have long been the gold standard for targeted protein quantification [18]. However, their multiplexing capacity is often limited to dozens of proteins [19]. Furthermore, they can be susceptible to cross-reactivity, leading to non-specific signals [20]. Olink’s PEA technology fundamentally enhances specificity through its dual-recognition requirement, where a signal is generated only when two distinct antibodies bind to the same target protein in close proximity [9, 10]. This, combined with its capacity to measure thousands of proteins from a minimal sample volume (~1 µL), represents a significant leap forward in scale and data quality [21].

Versus Aptamer-based Platforms (e.g., SomaScan): SomaScan, which uses modified DNA aptamers (Slow Off-rate Modified Optamers, SOMAmers) as affinity reagents, offers even broader proteome coverage (7000+ proteins). While powerful for discovery, aptamer-based platforms can sometimes exhibit different binding affinities and specificities compared to antibody-based methods, leading to discrepancies in quantification for certain proteins [22]. Olink’s reliance on well-validated monoclonal or polyclonal antibodies often aligns more closely with results from traditional immunoassays, which can facilitate the translation of findings into established clinical laboratory formats [23, 24].

In summary, Olink proteomics occupies a strategic niche, balancing the broad discovery potential of MS with the high specificity and sensitivity of immunoassays. This combination makes it exceptionally well-suited for cardiovascular research, where the precise quantification of signaling proteins across large patient populations is paramount for biomarker discovery and mechanistic insight.

## 3. Clinical Applications in Heart Failure Biomarker Discovery

### 3.1 Population-Based Screening and Risk Prediction

Large-scale epidemiological studies have leveraged Olink proteomics to identify circulating proteins predictive of incident HF, providing crucial insights into subclinical disease processes. The landmark investigation by Stenemo and colleagues [25] analyzed 80 cardiovascular proteins in elderly community-dwelling individuals, identifying nine proteins independently associated with future HF development. Growth differentiation factor-15 (GDF-15) and T-cell immunoglobulin mucin domain-1 (TIM-1) emerged as the strongest predictors, with hazard ratios exceeding 2.0 per standard deviation increase, surpassing traditional risk factors in predictive accuracy [26].

These findings have been validated and extended across diverse populations. Analysis of the Framingham Heart Study offspring cohort using expanded Olink panels identified additional proteins associated with incident HF, including markers of inflammation (IL-6, TNF-R1), vascular remodeling (MMP-2, MMP-9), and metabolic dysfunction (Fibroblast Growth Factor-23, FGF-23, leptin) [27]. Integration of proteomic profiles with clinical risk scores improved discrimination for HF prediction, with C-statistics increasing from 0.73 to 0.82 and net reclassification improvements exceeding 20%.

The temporal dynamics of protein changes preceding HF onset provide insights into disease evolution. Longitudinal proteomic profiling revealed that certain proteins, particularly GDF-15 and N-terminal pro-B-type Natriuretic Peptide (NT-proBNP), begin rising 5–10 years before clinical diagnosis, while others show accelerated increases in the year preceding symptom onset [25]. These temporal patterns suggest opportunities for staged intervention strategies based on proteomic risk profiles.

### 3.2 Biomarker Discovery in Acute Myocardial Infarction and Post-MI Heart Failure

Acute myocardial infarction (AMI) represents a critical precipitant of HF, with post-MI remodeling determining long-term outcomes. Olink proteomics has identified novel predictors of post-MI HF development and mortality. Skau and colleagues [28] utilized Olink panels to analyze 92 biomarkers in AMI patients, demonstrating that GDF-15 and TNF-related apoptosis-inducing ligand receptor 2 (TRAIL-R2) were the most powerful predictors of long-term all-cause mortality. Combination of these markers with traditional risk factors enabled effective discrimination between survivors and non-survivors, with area under the curve values approaching 0.85.

The technology has revealed distinct protein trajectories during post-MI recovery, with divergent patterns distinguishing patients who develop HF from those with preserved ventricular function [29]. Early elevation of proteins reflecting cardiomyocyte injury (troponins, heart-type fatty acid-binding protein (H-FABP)), inflammation (IL-6, c-reactive protein (CRP)), and extracellular matrix remodeling (matrix metalloproteinases (MMPs), tissue inhibitors of metalloproteinases (TIMPs)) characterized patients progressing to HF. Serial sampling demonstrated that persistence of inflammatory activation beyond 72 hours post-MI strongly predicted adverse remodeling and HF development.

Integration of proteomic data with cardiac imaging has enhanced understanding of post-MI remodeling mechanisms [30]. Proteins associated with specific imaging parameters include MMP-9 with left ventricular dilation, galectin-3 with myocardial fibrosis, and suppression of tumorigenicity 2 (ST2) with wall motion abnormalities. These protein-imaging correlations provide mechanistic links between circulating biomarkers and cardiac structural changes, informing therapeutic targeting strategies.

### 3.3 Clinical Implementation Workflows and Standardization

Translation of Olink proteomics into clinical practice requires standardized workflows encompassing pre-analytical, analytical, and post-analytical phases [17, 23, 31]. Pre-analytical considerations include sample collection protocols, processing timelines, and storage conditions. Studies have demonstrated that delayed processing beyond 4 hours at room temperature significantly affects certain protein measurements, particularly cytokines and growth factors. Standardized protocols recommend immediate centrifugation and storage at –80 °C to maintain protein stability.

Analytical standardization involves calibration procedures, quality control metrics, and batch effect correction [17, 23]. Multi-center studies have established inter-laboratory coefficients of variation below 20% for most proteins when following standardized protocols. Implementation of bridging samples and normalization algorithms has improved cross-study comparability, essential for establishing universal reference ranges and clinical decision thresholds.

Post-analytical challenges include data interpretation, clinical reporting, and integration with electronic health records [31]. Development of clinical decision support systems incorporating proteomic data with traditional risk factors has shown promise in pilot implementations. However, regulatory approval, reimbursement considerations, and clinician education remain barriers to widespread adoption.

### 3.4 Comparative Analysis of Traditional and Novel Biomarkers

The clinical utility of biomarkers discovered via proteomics must be evaluated against established standards, particularly natriuretic peptides. Traditional risk assessment in HF relies heavily on NT-proBNP, with well-established cutoffs for diagnosis and prognosis [12, 32]. However, NT-proBNP has known limitations, including its dependency on age and renal function, reduced levels in obesity, and limited ability to illuminate the specific pathophysiology driving an individual’s disease [33, 34, 35, 36].

Proteomic platforms like Olink have been instrumental in identifying markers that overcome these limitations by representing distinct biological pathways complementary to the myocardial stretch indicated by NT-proBNP. For example, ST2, a member of the interleukin-1 receptor family, has emerged as a key marker of myocardial fibrosis and inflammation [37, 38]. In acute HF, serial ST2 measurements predict outcomes independent of NT-proBNP changes, and the combination of both markers provides superior risk stratification [25, 39]. Similarly, galectin-3 (Gal-3), another protein involved in inflammation and fibroblast activation, offers additive prognostic value, further reinforcing the importance of assessing the fibro-inflammatory axis in HF [26, 40, 41, 42].

Beyond this axis, proteomics has consistently highlighted GDF-15 as one of the most powerful prognostic markers in HF [25, 27]. Reflecting a broad spectrum of cellular stress, including inflammation and mitochondrial dysfunction, elevated GDF-15 levels are strongly associated with mortality across both Heart Failure with reduced Ejection Fraction (HFrEF) and heart failure with preserved ejection fraction (HFpEF), often outperforming other established markers [43, 44, 45, 46]. Proteomic panels also enable a dynamic assessment of extracellular matrix remodeling through the simultaneous measurement of matrix metalloproteinases (MMPs) and their tissue inhibitors (TIMPs), providing a direct window into the structural changes underpinning adverse remodeling [47].

Furthermore, proteomics offers the sensitivity to refine the interpretation of established markers. While high-sensitivity cardiac troponins are central to diagnosing acute coronary syndromes, their utility in chronic HF has been less clear [48]. Olink-based studies have revealed that even minute troponin concentrations, previously considered clinically insignificant, are associated with increased long-term HF risk in community populations, prompting a re-evaluation of its role in subclinical disease detection. This multi-marker perspective, encompassing pathways of stretch, inflammation, fibrosis, cellular stress, and myocyte injury, provides a far more comprehensive risk profile than any single biomarker alone. However, it is crucial to temper this potential with the recognition that the incremental prognostic value of adding a large panel of markers to a robust clinical model that already includes NT-proBNP and key clinical variables is often modest and must be rigorously validated in diverse, large-scale cohorts before clinical implementation.

## 4. Mechanistic Insights and Pathophysiological Understanding

### 4.1 Molecular Networks in Heart Failure Development

Olink proteomics has fundamentally advanced understanding of molecular networks underlying HF pathogenesis. Network analysis of proteomic data reveals highly interconnected protein modules corresponding to distinct biological processes [32, 49, 50]. Central hub proteins, including GDF-15, ST2, and TIM-1, serve as key nodes linking inflammation, fibrosis, and metabolic dysfunction. These hub proteins show the strongest associations with clinical outcomes and represent potential therapeutic targets.

The technology has elucidated temporal evolution of molecular networks during HF progression [51, 52]. Early disease stages are characterized by activation of adaptive responses, including proteins involved in cellular stress response and tissue repair. Transition to maladaptive remodeling involves shift toward pro-inflammatory and pro-fibrotic protein signatures. Advanced HF exhibits dysregulation across multiple protein networks, reflecting systemic consequences of cardiac dysfunction.

Integration of proteomic data with genomic information has identified genetically regulated proteins contributing to HF susceptibility [53]. Mendelian randomization studies using Olink data have established causal relationships for several proteins, including IL-6 receptor and proprotein convertase subtilisin/kexin type 9 **(**PCSK9), validating them as therapeutic targets. These findings demonstrate the power of proteogenomics in distinguishing causal mediators from biomarkers of disease.

### 4.2 Inflammation and Immune Activation

Comprehensive proteomic profiling has revealed the central role of inflammation in HF pathogenesis, with distinct inflammatory signatures characterizing different etiologies and stages [39]. Analysis using Olink inflammation panels demonstrates activation of multiple inflammatory pathways, including acute-phase response, complement cascade, and cellular immunity. Temporal profiling during acute decompensation shows early elevation of damage-associated molecular patterns followed by sustained cytokine activation [54].

The technology has identified novel inflammatory mediators not previously implicated in HF. Proteins such as CD84 molecule (CD84) and TNFRSF10A, discovered through unbiased proteomic screening, show stronger associations with outcomes than traditional inflammatory markers [10]. These findings have expanded understanding of inflammatory mechanisms beyond classical cytokines to include cellular adhesion molecules, chemokines, and immune checkpoint proteins.

Studies in specific populations have revealed unique inflammatory profiles. HIV-associated cardiomyopathy exhibits distinct proteomic signatures reflecting chronic immune activation and accelerated atherosclerosis [55, 56]. Proteomic analysis of statin effects in HIV patients demonstrated modulation of six proteins involved in immune pathways, suggesting mechanisms for cardiovascular benefit beyond lipid lowering [56].

### 4.3 Cardiorenal Interactions and Systemic Effects

The cardiorenal syndrome represents a critical complication in HF, with bidirectional interactions between cardiac and renal dysfunction [57, 58]. Olink proteomics has identified protein signatures reflecting heart-kidney crosstalk, with kidney injury molecule-1 (KIM-1) emerging as a powerful predictor of adverse outcomes in patients with combined cardiac and renal dysfunction [59]. Longitudinal profiling reveals that kidney-derived proteins begin rising before traditional markers of renal function, suggesting opportunities for earlier intervention.

Analysis of 80 circulating proteins in cohorts with declining renal function identified multiple proteins associated with estimated glomerular filtration rate (eGFR) decline and incident chronic kidney disease [60]. TNF-receptor superfamily members, including TRAIL-R2 and CD40 Ligand (CD40L) receptor, showed the strongest associations, implicating inflammatory pathways in cardiorenal syndrome progression. These findings have informed development of anti-inflammatory strategies targeting shared pathophysiological mechanisms.

FGF-23 has emerged as a key mediator linking mineral metabolism disturbances with cardiovascular outcomes [58]. Proteomic studies demonstrate that FGF-23 elevation precedes phosphate abnormalities and associates with left ventricular hypertrophy independent of traditional risk factors. The identification of FGF-23 as both biomarker and potential therapeutic target exemplifies the translational potential of proteomic discoveries.

### 4.4 Metabolic Dysfunction and Energetic Failure

HFpEF exhibits prominent metabolic dysfunction, revealed through integrated proteomic and metabolomic profiling [59, 61, 62]. Olink cardiometabolic panels identify dysregulation of proteins involved in insulin signaling, adipokine regulation, and mitochondrial function. Key findings include elevated Insulin-like Growth Factor-Binding Protein 1 (IGFBP-1) reflecting insulin resistance, altered adiponectin indicating adipose tissue dysfunction, and increased GDF-15 suggesting mitochondrial stress.

The relationship between diabetes and HF has been elucidated through proteomic analysis of diabetic cardiomyopathy [63, 64]. Oxidative stress pathways show particular prominence, with proteins reflecting reactive oxygen species production and impaired antioxidant defenses. These findings have identified potential therapeutic targets for preventing diabetic HF, including pathways amenable to pharmacological modulation.

Proteomic profiling has revealed metabolic differences between HF phenotypes [62, 65]. HFrEF exhibits alterations in proteins related to glucose utilization and fatty acid oxidation, reflecting metabolic shift from fatty acids to glucose. HFpEF shows more pronounced abnormalities in proteins regulating lipid metabolism and adipokine signaling, consistent with metabolic syndrome association. These phenotype-specific metabolic signatures suggest tailored therapeutic approaches targeting distinct metabolic pathways.

### 4.5 Cell Death Pathways and Tissue Remodeling

Olink proteomics has provided insights into cell death mechanisms and tissue remodeling processes in HF [61, 66]. Analysis of apoptosis-related proteins reveals activation of both intrinsic and extrinsic cell death pathways, with Fas, TNF-R1, and TRAIL-R2 showing strong associations with disease severity. Temporal profiling during acute decompensation demonstrates waves of cell death marker release, suggesting ongoing myocardial injury even after clinical stabilization.

Studies in systemic lupus erythematosus patients with cardiovascular complications have revealed enhanced apoptotic signaling, with higher levels of death receptors in those with cardiac involvement [66]. These findings suggest that autoimmune mechanisms may contribute to myocardial injury through enhanced susceptibility to apoptosis, providing rationale for immunomodulatory therapy in selected patients.

Matrix remodeling proteins show dynamic changes throughout HF progression [67, 68]. Early elevation of MMP-2 and MMP-9 reflects adaptive remodeling, while later increases in tissue inhibitors of metalloproteinases indicate transition to fibrosis. The balance between matrix degradation and synthesis proteins provides insights into remodeling phenotypes and potential therapeutic windows for anti-fibrotic interventions.

## 5. Technical Advances and Methodological Innovations

### 5.1 Platform Evolution and Expanded Capabilities

The Olink platform has undergone continuous evolution, with recent advances substantially expanding analytical capabilities [47, 67, 69]. Development of Explore panels now enables simultaneous measurement of over 5000 proteins, approaching comprehensive proteome coverage. Technical improvements include enhanced antibody validation procedures, optimized oligonucleotide designs reducing background signal, and improved normalization algorithms minimizing batch effects.

Novel applications have extended beyond traditional plasma/serum analysis. Single-cell proteomics combining Olink with flow cytometry enables simultaneous measurement of surface and intracellular proteins at single-cell resolution [47]. This capability has revealed cellular heterogeneity in circulating immune cells from HF patients, identifying distinct activation states associated with disease severity. Spatial proteomics applications allow mapping protein expression within tissue sections, providing insights into regional heterogeneity in failing hearts.

Integration with other molecular profiling technologies has created multi-dimensional datasets [47]. Combined RNA and protein analysis at single-cell level reveals post-transcriptional regulation in HF, identifying discordances between mRNA and protein levels for key molecules. These integrated approaches provide more complete understanding of molecular mechanisms than either technology alone.

### 5.2 Comparative Analysis With Alternative Proteomic Platforms

While Olink has gained prominence, comparison with alternative proteomic platforms provides perspective on relative strengths and limitations [53, 70]. Mass spectrometry-based proteomics offers unbiased discovery potential and ability to detect post-translational modifications. However, plasma protein dynamic range remains challenging, with abundant proteins masking detection of low-abundance signaling molecules critical to HF pathophysiology.

Alternative multiplex immunoassay platforms, including Luminex and MesoScale Discovery, provide complementary capabilities [70]. Photonic crystal-enhanced fluorescence immunoassays combined with machine learning have shown promise for point-of-care applications, achieving good performance for NT-proBNP detection with simplified workflows. However, multiplexing capacity remains limited compared to Olink, restricting comprehensive profiling capabilities.

SomaScan, utilizing aptamer-based technology, represents another high-throughput platform with coverage exceeding 7000 proteins [53]. Comparative studies in cardiovascular cohorts show moderate correlation between platforms (r = 0.4–0.7), with platform-specific biases reflecting different capture reagents and detection principles. These differences emphasize the importance of platform selection based on specific research questions and validation using orthogonal methods.

### 5.3 Computational Approaches and Bioinformatics

The complexity of Olink-generated data necessitates sophisticated computational approaches for analysis and interpretation [32, 50]. Traditional statistical methods face challenges with high-dimensionality, multicollinearity, and multiple testing burden. Machine learning algorithms, including random forests, support vector machines, and neural networks, have proven valuable for biomarker selection and outcome prediction.

Network-based approaches reveal functional relationships between proteins, identifying disease modules and key regulatory nodes [49]. Weighted gene co-expression network analysis adapted for proteomics identifies protein modules associated with clinical traits. These modules often correspond to biological pathways, providing functional interpretation of complex protein signatures. Integration with protein-protein interaction databases enhances understanding of molecular mechanisms.

Deep learning applications show particular promise for pattern recognition in longitudinal proteomic data [50, 71, 72]. Recurrent neural networks can model temporal protein dynamics, predicting future trajectories based on baseline profiles. These predictive models enable identification of patients at risk for adverse outcomes before clinical deterioration, supporting proactive intervention strategies.

### 5.4 Quality Control and Standardization Initiatives

Ensuring data quality and reproducibility requires rigorous quality control procedures throughout the analytical workflow [17, 23]. Pre-analytical standardization includes detailed protocols for sample collection, processing, and storage. Implementation of standard operating procedures across participating sites has reduced pre-analytical variation, with coefficients of variation below 15% for most proteins in multi-center studies.

Analytical quality control involves multiple checkpoints, including assessment of technical replicates, monitoring of internal controls, and evaluation of batch effects [23]. Statistical process control charts track assay performance over time, enabling early detection of analytical drift. Implementation of bridging samples between batches allows robust normalization, essential for longitudinal studies and meta-analyses.

Post-analytical standardization focuses on data processing and reporting [17]. Development of consensus pipelines for data normalization, missing value imputation, and outlier detection has improved cross-study comparability. Establishment of reference ranges in healthy populations provides context for clinical interpretation, though population-specific considerations remain important.

## 6. Clinical Studies and Therapeutic Applications

### 6.1 Heart Failure Phenotyping and Classification

Olink proteomics has revolutionized HF phenotyping, revealing molecular heterogeneity within traditional classifications [11, 49]. Analysis comparing HFrEF and HFpEF demonstrates overlapping yet distinct proteomic signatures. While 70% of dysregulated proteins are shared, phenotype-specific patterns provide insights into differential pathophysiology. HFrEF shows prominent neurohormonal activation markers, while HFpEF exhibits metabolic and inflammatory predominance.

The presence of comorbidities further modifies proteomic profiles [11]. Atrial fibrillation in HFrEF associates with enhanced inflammatory signaling, while in HFpEF, the association is attenuated, suggesting different pathophysiological contributions. Diabetes amplifies metabolic protein dysregulation particularly in HFpEF, supporting distinct therapeutic approaches based on comorbidity profiles.

Machine learning applied to proteomic data has identified novel HF subphenotypes not apparent from clinical parameters alone [49]. Unsupervised clustering reveals groups with distinct protein signatures, clinical trajectories, and treatment responses. These molecularly-defined subgroups show better prognostic discrimination than traditional classifications, supporting transition toward precision medicine approaches.

### 6.2 Therapeutic Target Identification and Drug Development

Proteomic discoveries have identified novel therapeutic targets and elucidated mechanisms of existing medications [56, 73, 74, 75]. Analysis of sacubitril/valsartan effects reveals suppression of proteins involved in myocardial stress and fibrosis while enhancing cardioprotective pathways [73]. These mechanistic insights extend beyond expected neprilysin inhibition effects, suggesting pleiotropic benefits contributing to clinical efficacy.

Sodium-glucose cotransporter 2 (SGLT2) inhibitors, originally developed for diabetes, show remarkable cardiovascular benefits revealed through proteomic profiling [75]. Treatment associates with changes in proteins regulating erythropoiesis, iron metabolism, and cellular energy metabolism. These unexpected findings have prompted investigation of SGLT2 inhibitors in non-diabetic HF, with clinical trials confirming benefit across the glycemic spectrum.

Novel therapeutic targets identified through Olink proteomics are entering clinical development [74]. Proteins validated through Mendelian randomization as causal mediators represent particularly attractive targets. Early-phase trials incorporating proteomic biomarkers for patient selection and pharmacodynamic assessment show improved success rates, supporting biomarker-guided drug development strategies.

### 6.3 Clinical Trial Applications and Biomarker-Guided Therapy

A primary goal of biomarker discovery is to inform clinical decision-making, either by guiding therapy or by refining patient selection in clinical trials. The concept of “biomarker-guided therapy”—where treatment is titrated to a biomarker target—has been a long-standing aspiration in HF, though its path has been challenging. Early efforts targeting natriuretic peptides, culminating in the large-scale guiding evidence based therapy using biomarker intensified treatment in heart failure (GUIDE-IT) trial, failed to demonstrate clinical benefit over standard care, teaching the field that targeting a general marker of hemodynamic stress may be insufficient [76]. Subsequent trials, such as systemic microvascular endothelial and coronary epicardial adipose tissue pRoteomics in heart failure (SECRET-HF) using ST2 to guide mineralocorticoid receptor antagonist (MRA) therapy, represented a conceptual advance by targeting a more specific pathway (fibrosis/inflammation), though this trial also did not meet its primary endpoint [77].

These experiences have prompted a paradigm shift: from using biomarkers to guide therapy titration to using them for patient stratification. Olink proteomics has become a powerful tool in this new approach, particularly in the post-hoc analysis of major clinical trials. For example, comprehensive proteomic profiling within the prospective comparison of ARNI with ACEI to determine impact on global mortality and morbidity in heart failure (PARADIGM-HF) and prospective comparison of ARNI with ARB global outcomes in heart failure with preserved ejection fraction (PARAGON-HF) trials revealed that baseline protein signatures could predict differential treatment effects [78, 79]. Patients with higher levels of inflammatory and fibrotic markers at baseline appeared to derive greater benefit from sacubitril/valsartan, suggesting that these proteomic profiles could potentially identify patient subgroups most responsive to specific therapies in the future [61, 75]. This application—moving beyond prognosis to predict therapeutic response—is a critical step toward personalized medicine.

Looking forward, the ultimate goal is to integrate these insights into prospective, adaptive trial designs. Olink-derived proteomic signatures could be used at the screening phase to enrich trial populations with patients most likely to respond, or to stratify randomization based on underlying pathophysiology (e.g., an “inflammatory HFpEF” vs. a “fibrotic HFpEF” subtype). Furthermore, serial on-treatment monitoring of specific protein trajectories could identify early signals of efficacy or target engagement, allowing for more efficient dose-finding or proof-of-concept studies [61]. While pilot programs implementing proteomic-guided HF management in real-world settings show promise, large-scale randomized trials designed with a priori biomarker stratification are now the critical next step to definitively establish the clinical utility of this approach and translate the wealth of proteomic data into tangible patient benefits [73, 75].

### 6.4 Special Populations and Personalized Medicine

Proteomic profiling in special populations reveals unique considerations for personalized medicine approaches [12, 24]. Pediatric HF exhibits distinct protein signatures reflecting developmental differences and etiology distribution. Growth factors and developmental proteins show prominent dysregulation, suggesting age-specific therapeutic targets. Translation of adult biomarkers to pediatric populations requires careful validation given developmental proteome changes.

Sex-specific differences in the plasma proteome have important implications for HF management [24]. Women show lower baseline levels of cardiac injury markers but higher inflammatory proteins. These differences contribute to sex-specific HF presentations and outcomes. Proteomic studies reveal that women may benefit from different therapeutic approaches, particularly for HFpEF where female predominance suggests distinct pathophysiology.

Ethnic variations in protein levels and disease associations challenge universal biomarker application [24]. Studies in diverse populations reveal substantial differences in baseline proteomes and protein-outcome relationships. Development of population-specific reference ranges and risk models is essential for equitable precision medicine implementation. Multi-ethnic cohort studies using Olink proteomics are addressing these gaps.

## 7. Controversies, Challenges, and Future Directions

### 7.1 Barriers to Clinical Adoption and Implementation

Despite its immense research potential, the translation of Olink proteomics from discovery platforms to routine clinical tools faces significant and multifaceted barriers.

A primary hurdle is practical and economic. The substantial cost associated with high-plex proteomic assays remains prohibitive for widespread clinical use outside of well-funded research settings [80]. Moreover, the workflow is currently centralized in specialized laboratories, leading to long turnaround times incompatible with the rapid decision-making required in acute clinical scenarios, such as in the emergency department [81, 82]. Until cost-effective, rapid platforms are developed, proteomics will likely remain a tool for risk stratification in stable outpatient settings rather than for acute diagnosis [70].

Analytical and pre-analytical standardization presents another major challenge. While Olink has made strides in platform consistency, significant issues persist that hinder the establishment of universal clinical reference ranges. Inter-laboratory variability, a lack of certified reference materials for hundreds of proteins, and the profound impact of pre-analytical variables—such as sample type, anticoagulant use, and processing times—can all introduce biases that complicate the development of robust clinical decision cutoffs [17, 23].

The inherent biological and interpretive complexity of the data also complicates clinical translation. It is profoundly challenging to determine whether a circulating protein is a causal mediator or merely a bystander marker of disease processes [51, 52]. Furthermore, the influence of crucial comorbidities like chronic kidney disease and diabetes mellitus significantly alters the proteome, confounding the interpretation of an HF-specific signal. Disentangling these overlapping signals requires sophisticated analytical approaches that are not yet standard in clinical practice [83, 84].

Perhaps the most significant barrier, however, is the lack of definitive clinical utility and resulting physician inertia. A strong prognostic association, as demonstrated in countless studies, does not guarantee that acting on a biomarker will improve patient outcomes—a hard lesson learned from the GUIDE-IT trial [85, 86, 87]. Without Level 1 evidence from a randomized controlled trial showing clear benefit, clinicians are unlikely to adopt a complex and costly new test over established markers like NT-proBNP. Overcoming this “clinical inertia” will require not just demonstrating statistical significance, but proving clear, simple, and actionable value, complete with validated cut-points and interpretive guidelines that can be seamlessly integrated into existing clinical workflows.

### 7.2 Future Technological Developments

Next-generation Olink platforms promise expanded capabilities and improved performance [32, 50]. Development of panels approaching whole-proteome coverage will enable truly unbiased discovery. Technical advances including improved antibody engineering, enhanced signal amplification, and novel detection methods will further increase sensitivity and specificity.

Integration with other omics technologies will provide comprehensive molecular characterization [50]. Combined proteomic, genomic, transcriptomic, metabolomic, and epigenomic profiling will reveal regulatory relationships and identify therapeutic targets. Single-cell multi-omics will dissect cellular heterogeneity in HF, revealing cell type-specific pathological mechanisms.

Point-of-care proteomic devices are under development, potentially enabling rapid bedside testing [70]. Miniaturized platforms using microfluidics and smartphone-based detection could democratize access to proteomic profiling. However, maintaining analytical performance while reducing complexity remains challenging.

### 7.3 Artificial Intelligence and Precision Medicine Implementation

Artificial intelligence applications will transform proteomic data analysis and clinical implementation [50]. Machine learning algorithms can identify complex protein patterns invisible to traditional statistics. Deep learning models trained on large datasets achieve superior predictive performance for outcomes and treatment response.

Digital twin models incorporating proteomic profiles enable personalized risk prediction and treatment simulation [75]. These virtual patient representations predict individual responses to interventions, optimizing treatment selection. Integration with wearable devices and continuous monitoring will enable real-time risk assessment and proactive intervention.

Implementation of precision medicine requires healthcare system transformation [49]. Electronic health record integration, clinical decision support systems, and provider education are essential. Regulatory frameworks, reimbursement models, and ethical considerations regarding data privacy and equity must be addressed.

### 7.4 Multi-Omics Integration and Systems Medicine

The future of HF research lies in multi-omics integration within systems medicine frameworks [88, 89]. Combining proteomics with other molecular profiles provides comprehensive disease characterization. Network medicine approaches reveal disease modules and identify key regulatory nodes for therapeutic targeting [90].

Longitudinal multi-omics studies will elucidate disease trajectories and identify transition points [91, 92]. Understanding molecular changes preceding clinical transitions enables preventive interventions. Population-scale studies will reveal molecular subtypes and enable refined disease taxonomy.

International collaborations and data sharing initiatives will accelerate discovery [93]. Standardized protocols, common data models, and federated analysis platforms will enable mega-analyses. Cloud-based platforms will democratize access to analytical tools and facilitate collaboration.

### 7.5 Therapeutic Implications and Drug Development

Proteomic discoveries will drive next-generation therapeutic development [44, 92]. Validated protein targets are entering drug development pipelines. Biological therapies including antibodies and recombinant proteins offer precise targeting of dysregulated pathways [94].

Biomarker-guided trial designs will improve development efficiency [90]. Enrichment strategies based on proteomic profiles increase statistical power and reduce sample size requirements. Adaptive designs with proteomic endpoints enable rapid iteration and optimization [71, 95].

Companion diagnostics based on proteomic profiles will enable precision therapy. Treatment selection algorithms incorporating protein signatures will optimize individual outcomes. Serial monitoring will guide therapy adjustment and identify treatment resistance early.

## 8. Conclusions and Future Perspectives

Olink proteomics has catalyzed a paradigm shift in HF research, transitioning the characterization of this syndrome from a set of clinical and hemodynamic observations to a high-resolution molecular landscape. Its primary contribution lies in the robust identification of prognostic protein signatures that outperform traditional markers and reveal the profound heterogeneity within HF phenotypes, particularly HFpEF.

However, a critical gap persists between this prognostic power and demonstrable clinical utility. The field is now saturated with biomarkers that predict adverse outcomes, yet very few inform therapeutic decisions. The central challenge, therefore, is to pivot from correlation to causality—to distinguish proteins that are merely passengers of the disease state from those that are actionable drivers of pathophysiology.

Future progress will not be defined by discovering more biomarkers, but by their strategic deployment to answer key biological and clinical questions. The next frontier demands a rigorous focus on mechanistic validation, integrating proteomics with Mendelian randomization to establish causal pathways, with single-cell technologies to pinpoint cellular origins, and ultimately, with biomarker-stratified clinical trials. The success of proteomics will be measured not by the length of the biomarker list, but by its ability to guide interventions, identifying patient subgroups who uniquely benefit from therapies targeting specific protein-driven pathways.

In conclusion, Olink proteomics has revolutionized heart failure research, moving the field beyond single markers like NT-proBNP towards a multi-pathway understanding of the disease, incorporating insights from inflammatory, fibrotic, and metabolic axes. While proteomic signatures have demonstrated superior risk stratification and provided profound mechanistic insights, their translation into routine clinical practice faces significant hurdles, including cost, standardization, and the critical need for evidence from biomarker-stratified randomized controlled trials. Future developments in technology and the integration of AI hold immense promise, but overcoming these practical barriers will be the true determinant of proteomics’ ultimate impact on patient care. The journey from complex data to tangible clinical benefit is challenging, yet essential for realizing the vision of precision medicine in heart failure.
